# Diagnostic value of niacin skin blunting response in adolescent patients with depression

**DOI:** 10.1186/s12888-023-05294-7

**Published:** 2023-11-08

**Authors:** Shen Nianhong, Liu Pan, Li Caijun, Ye Hongying, Sun You, Chang Jie, Zhou Jinquan, Zhou Yunshan, Wang Donghu, Yu Mingchao, Huang Chengbing, Hou Xueyuan

**Affiliations:** 1 Department of Psychiatry, Huai’an Third People’s Hospital, No. 272, Huaihai West Road, Huai’an, 223001 China; 2 Department of Geriatric Psychiatry, The Affiliated Brain Hospital of Nanjing Medical University, Nanjing, 210029 China

**Keywords:** Skin flushing reaction, Depression, Adolescents

## Abstract

**Objective:**

To investigate the differences in the niacin skin flushing response of adolescent depressed patients and healthy adolescents and its diagnostic value in adolescent depression.

**Methods:**

Thirty-eight cases of acute episodes of depression in unmedicated adolescents and 47 age- and sex-matched healthy controls were included as study subjects, and sociodemographic and clinical data were collected, all of which were stimulated with six concentration gradients (up to 60 mmol/L, followed by sequential 3-fold gradient dilution to a minimum of 0.25 mmol/L) of niacin solution on the forearm skin, and the skin flushing area was applied as an assessment index.

**Results:**

The total area of redness of the skin in response to niacin was significantly lower in the adolescent depression group than in the healthy adolescent group (Z=-3.36, p = 0.001) and was able to distinguish the adolescent depression group from the healthy adolescent group (area under curve = 0.713, sensitivity 51.1%, specificity 83.2%).

**Conclusions:**

Niacin sensitivity is reduced in adolescent depressed patients, and the niacin skin flush response has potential clinical value as a diagnostic biomarker for adolescent depression.

## Background

Adolescence is a critical period of neurological development and characterizes the rapid development of cognitive, emotional, and social functioning and mental health during this stage [1]. Therefore, adolescents are prone to emotional distress and are the age group with a high prevalence of depression. Epidemiological surveys have shown that 11-28% of adolescents experienced at least one episode of major depression by the age of 19 years, with a median age of onset of 15.5 years [2, 3]. In its most severe form, depression may lead to suicide attempts or suicidal behavior, which is one of the leading causes of death in adolescents. However, unlike adult depression, adolescent depression has atypical symptoms and is associated with adverse transitions to adulthood, such as depression, anxiety, substance abuse, and domestic violence. As there are no independent diagnostic criteria for adolescent depression, only adult depression criteria are used, and there is a lack of clinical biological markers, so the opportunity for early detection, early diagnosis, and early treatment is very easy to miss [4].

Niacin, a B vitamin, has been suggested to be closely associated with the development of depression [5]. Niacin acts on the skin and can be activated through the arachidonic acid-cyclooxygenase pathway, which can cause skin flushing symptoms, called niacin skin flushing reaction, which can indirectly respond to the level of oxidative stress in the body and has potential auxiliary diagnostic value in psychiatric disorders. Messamore E reported that the niacin response biomarker may be as a schizophrenia endophenotype [6]. And niacin skin blunting response has high diagnostic value for in adolescent patients with schizophrenia or bipolar [7].niacin skin sensitivity is increased in adolescents risk for Psychosis [8]. These findings suggest that altered niacin sensitivity may bemore relevant for the onset of psychosis.

Previous studies suggest that blunted niacin skin response may be one of the phenotypic features of depression in adult depressed patients.One study reported a negative correlation between niacin sensitivity and the severity of symptoms and a significant association of flushing deficits with depressed mood, feelings of anxiety, and somatic symptoms (loss of appetite and weight loss) [9]. However, findings pertinent to depression have been inconsistent, with some studies suggesting no difference in niacin tidal response in depression compared to healthy controls [10]. However, in recurrent depression, some studies have suggested an enhanced niacin skin flush response in depressed patients [11]. Only a few studies have been conducted on the niacin skin response in affective disorders, and to our knowledge, there are no reports on its application in adolescent depression. Therefore, this study intended to investigate the value of niacin skin response in adolescent depression and provide possible biomarkers for the diagnosis of adolescent depression in terms of oxidative stress.

### Subjects and methods

#### Study subjects

**Fig. 1:**
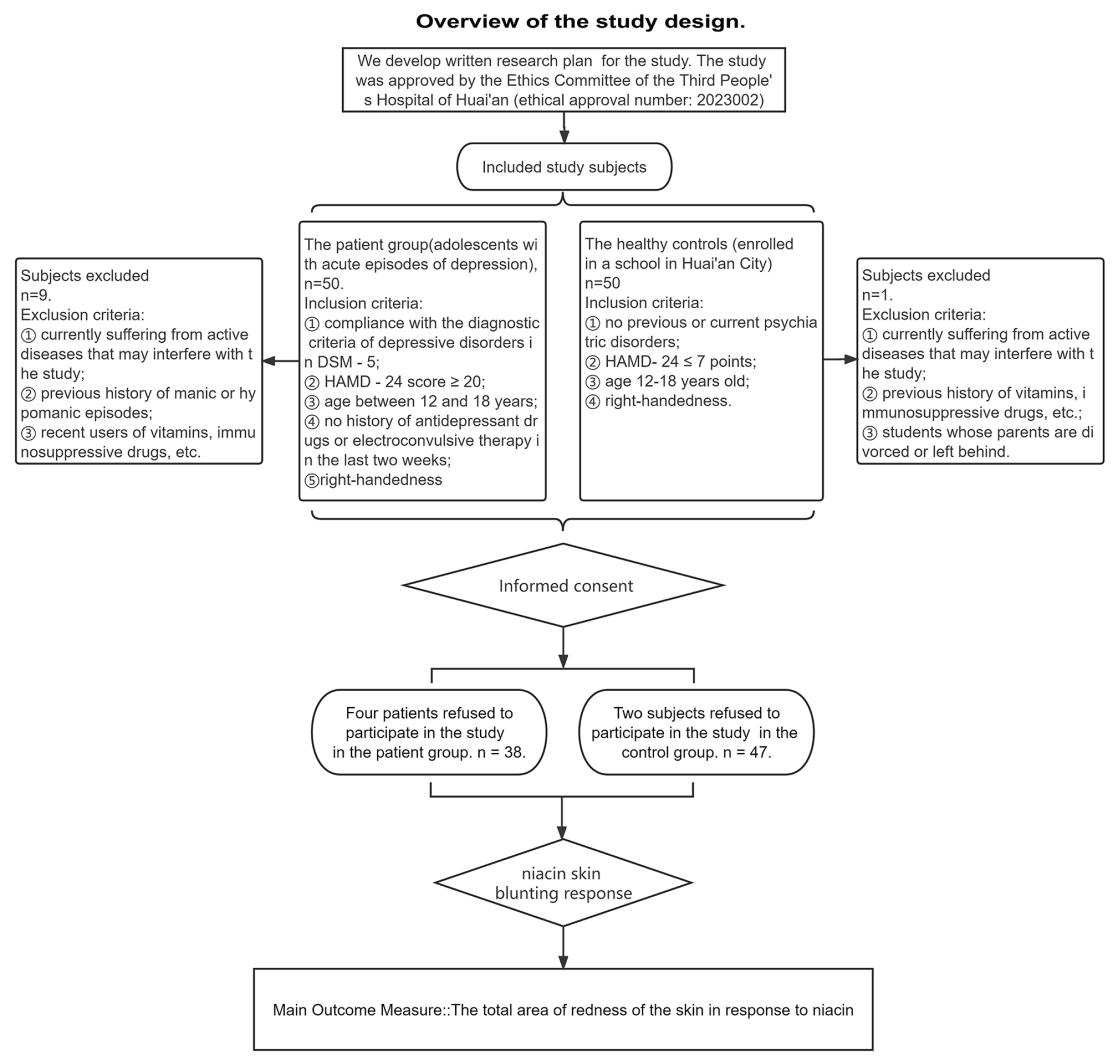
Overview of the study design. The workfow schematic diagram for this study illustrates the details of data collection

The outline of our research design is shown in the flow chart in Fig. 1. The patient group comprised those with acute episodes of adolescent depression who were hospitalized in Huai’an Third People’s Hospital in April 2021. Inclusion criteria were: ① compliance with the diagnostic criteria of depressive disorders in the Diagnostic and Statistical Manual of Mental Disorders, fifth edition (DSM − 5); ② Hamilton depression scale 24-item (HAMD − 24) score ≥ 20; ③ age between 12 and 18 years; ④ no history of antidepressant drugs or electroconvulsive therapy in the last two weeks; ⑤ The skin reaction of the right arm of different handedness groups may be different, so in order to reduce confounding factors as much as possible, both the patient group and the control group were selected to be right-handed. Exclusion criteria were: ① current active diseases that may interfere with the study (e.g., fever, immune diseases such as systemic lupus erythematosus, endocrine diseases such as hyperthyroidism, and nutritional diseases such as vitamin deficiency); ② previous history of manic or hypomanic episodes; ③ recent users of vitamins, immunosuppressive drugs, etc.

Healthy controls were from students enrolled in a school in Huai’an City. Inclusion criteria were: ① no previous or current psychiatric disorders; ② HAMD-24 ≤ 7 points; ③ age 12–18 years old; ④ right-handedness. Exclusion criteria: ① currently suffering from active diseases that may interfere with the study (e.g., immune diseases such as fever, systemic lupus erythematosus, endocrine diseases such as hyperthyroidism, and nutritional diseases such as vitamin deficiency); ② those who have recently taken vitamins, immunosuppressive drugs, etc.; ③ students whose parents are divorced or left behind.

The study was approved by the Ethics Committee of the Third People’s Hospital of Huai’an (ethical approval number: 2,023,002), and all subjects signed the informed consent form.

## Methods

### Collection of demographic and clinical data

A homemade scale was used to collect general information about the subjects, and disease information such as the patients’ age of first onset, number of onsets, course of diagnosis and treatment, and duration of the current disease. The HAMD-24 and Hamilton anxiety scale (HAMA) were used to assess the clinical symptoms and the severity of the disease.

### Niacin skin flushing reaction test

The niacin skin test and the flushing assessment were performed on the BrainAid Skin Niacin Response Test Instrument TY-AraSnap-H100 (Shanghai Tianyin Biological Technology Ltd., Shanghai, China). This test was performed by referring to the Niacin skin reaction operation method described by G.E. Berger et al. [8]. Filter paper sheets of 0.9 cm diameter were immersed in six different concentrations of niacin solution (up to 60 mmol/L, then diluted in a 3-fold gradient to a minimum of 0.25 mmol/L), and then the filter paper sheets dipped from low to high concentration of niacin solution were pasted on the subject’s inner forearm from the wrist to the elbow in turn for 1 min; the filter paper sheets were then removed, and the subjects were photographed and recorded every 10 s. The skin redness was also recorded every 10 s until 10 min.

### Statistical methods

The data were analyzed by SPSS20, and the categorical data, such as gender, were described by the chi-square test. Data expressed as median [first quartile; third quartile] or median ± SE. Differences between normally distributed continuous data were analyzed using parametric tests, and data that were not normally distributed were analyzed using non-parametric tests, using the receiver operating characteristic (ROC) curve to evaluate the total area of redness on depression. The diagnostic efficacy of the ROC was evaluated, and the diagnostic threshold was determined according to the highest threshold of the Jorden index, and the sensitivity and specificity were calculated at this threshold.

## Results

### General information

Thirty-eight adolescents (13 males and 25 females) with depression, aged 14.50 ± 1.45 years, and 47 healthy adolescent students (20 males and 27 females), aged 14.70 ± 1.10 years, were included. There were no statistical differences between the two groups in terms of age and gender (P > 0.05)(details in Table 1).

**Table 1:** Sample Characteristics of two group of subjects (N = 85)

		MDD group (n = 38)	HC group (n = 47)	X^2^, t or Z	*p*
Gender	male	13	20	0.616	0.433
	female	25	27		
Age	(year)	14.50 ± 1.45	14.70 ± 1.10	0.731	0.467
Duration of MDD	(month)	7 (4, 12)	—	—	—
Grade	7 th	8	14	0.877	0.645
	8 th	23	26		
	9 th	7	7		
HAMD−24 Scores		39.37 ± 2.48	1.96 ± 0.78	97.88	<0.001
HAMA Scores		23.87 ± 3.55	2.11 ± 0.60	41.36	<0.001
total skin area of niacin (mm^2^)		3021 (1446, 7776)	8933 (3625, 12,094)	−3.36	0.001

### Total area of skin erythema in response to niacin between the two groups

**Fig. 2:**
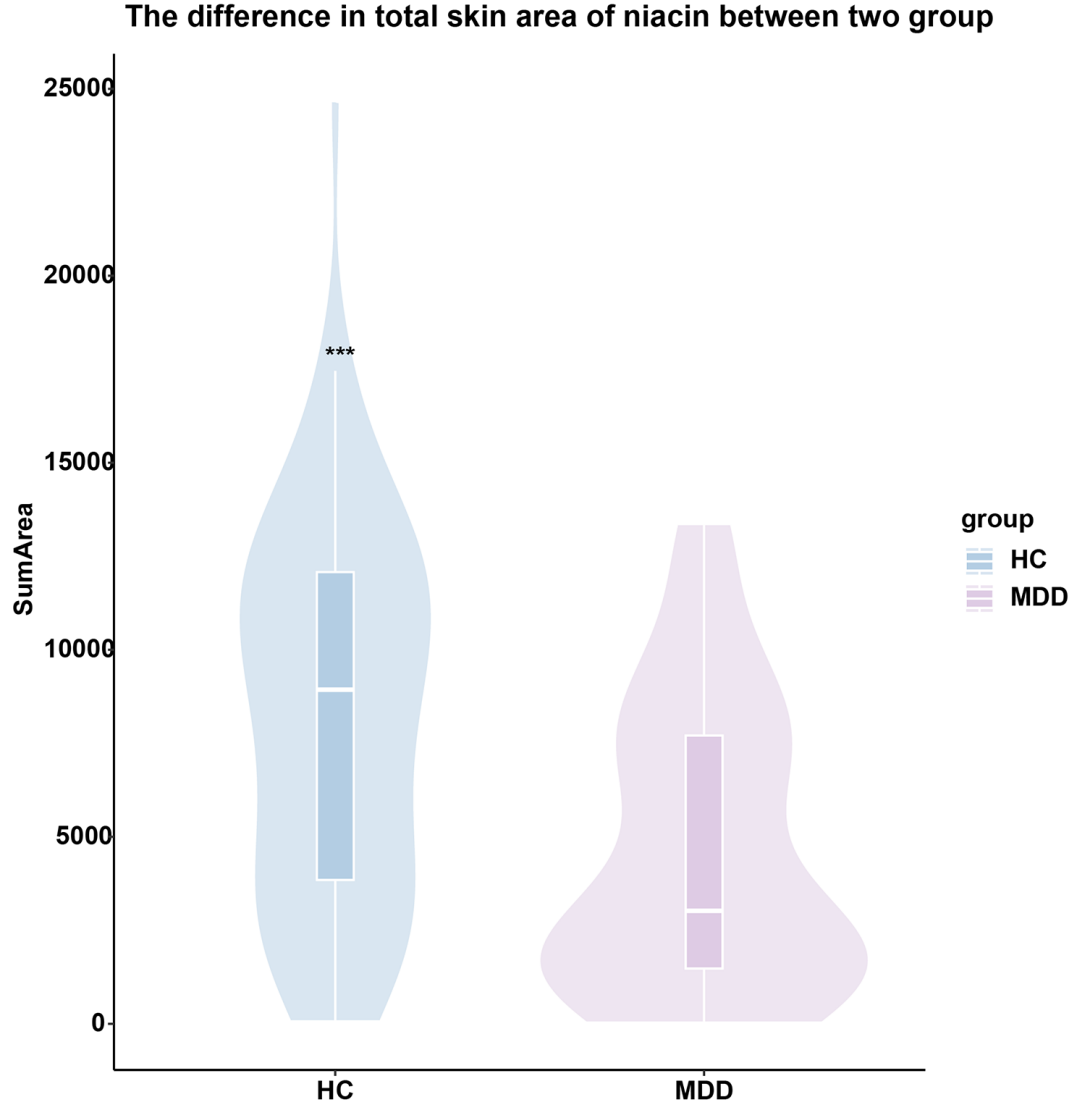
The difference in total skin area of niacin between the patient group and the control group

Comparison of niacin skin reactions in adolescents with confirmed depression (n = 38) vs. healthy adolescents (n = 47) revealed that the total niacin skin area was significantly lower in the adolescent depression group than in the healthy adolescents (Z=-3.36, p = 0.001) (See Fig. 2).

### The diagnostic value of niacin skin flushing in adolescent depression

**Fig. 3:**
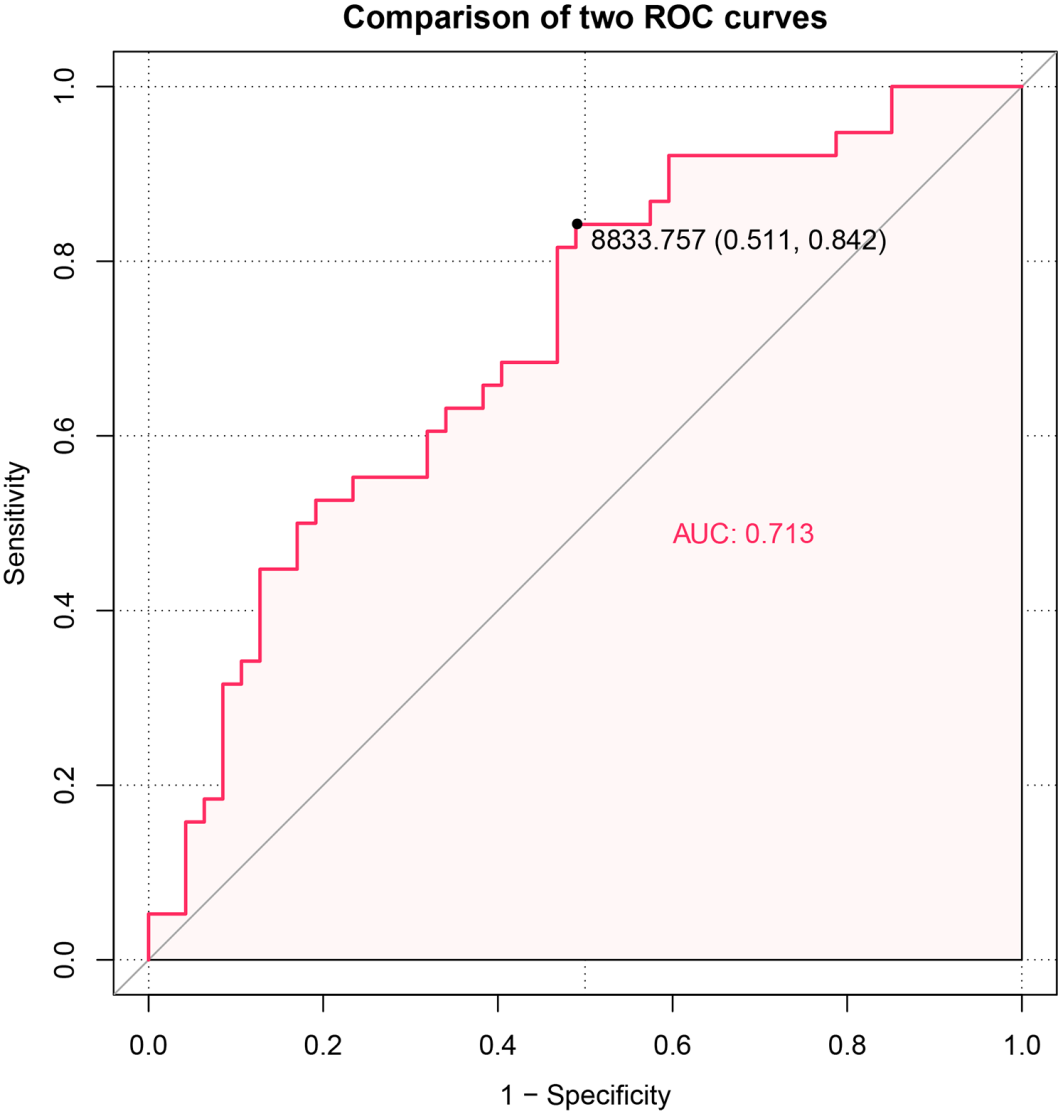
Receiver operating characteristic (ROC) curve of the total area value of skin flushing in response to niacin skin flushing on the effectiveness of depression differentiation

For the differentiation of depressed adolescents from healthy adolescents in terms of the total skin area of niacin response, the area under the curve (AUC = 0.713, P = 0.001, 95% CI 0.604–0.823), sensitivity was 51.1%, specificity was 83.2%, and the optimal threshold was 8689.12 mm^2^ (see Fig. 3).

## Discussion

Niacin is a water-soluble vitamin with a high affinity for its receptor, GPR109A [12], and produces specific anti-inflammatory and antioxidant effects associated with a variety of neurological pathologies, such as Parkinson’s disease, depression, and schizophrenia [13]. The flushing response of the skin to niacin is based on the binding of niacin to G protein-coupled receptors on epidermal Langerhans cells and stimulates the release of arachidonic acid (AA) from cell membrane phospholipids by phospholipase A2 (PLA2); the free AA generates prostaglandins with vasodilatory properties via cyclooxygenase, which in turn causes skin flushing [14]. In contrast, depression occurs in patients with abnormal activation of immune inflammatory and oxidative stress pathways, which can activate inflammatory pathways of phospholipid and arachidonic acid metabolism [15]. In recent years, an increasing number of studies have suggested that the skin flush response to niacin can be used as an indirect response to oxidative stress and inflammation levels in patients with depression by assessing niacin sensitivity through skin flush area and flush rate. However, it has been rarely applied to address adolescent depression; therefore, the adolescent population was selected for this study.

The present study shows that the total area of redness caused by niacin stimulation of the skin in adolescent depression was significantly smaller than in the healthy adolescent population (p = 0.001), consistent with findings in adult depression [11, 16]. However, no significant difference in skin flushing response between depressed patients and healthy adults has also been suggested [5]. The reason for this discrepancy may be the different ways of detecting the skin response to niacin and the different concentrations of niacin chosen, which is one of the limitations of the findings. However, the index relying on one concentration of niacin is often unstable, so the total area of redness caused by each concentration selected in this study was characterized as a response to niacin sensitivity and reduced the bias caused by one concentration. However, compared to the application of laser Doppler flowmetry to quantify the niacin skin flushing response, the photographic method was used, which has poor test reliability and is thus a shortcoming. Thus, the study results need to be extended with caution.

The study also determined the ability of the niacin skin response to discriminate between adolescent depression and healthy adolescents by calculating the area under the curve (AUC) of the (ROC) (AUC = 0.713, sensitivity 51.1%, specificity 83.2%). However, considering sensitivity and specificity, the use of niacin skin response alone as a differentiation tool is not yet achievable.

We found abnormalities in the niacin skin response in adolescent depressed patients, but the mechanism of this abnormality is not clear. However, it suggests the presence of reduced niacin sensitivity in adolescent depressed patients, and the current study suggests that a deficiency of 5-hydroxytryptamine (5-HT) leads to depression and that 5-HT can occupy 5HT-2 A, 2B, and 2 C receptors, which in turn activate PLA2, signaling the relevant receptors through the production of arachidonic acid metabolites [17]. Thus, depressed patients with reduced 5-HT activate less PLA2 when niacin is stimulated on the skin, and the dysfunction of the PLA2 signaling cascade may affect neurotransmitter production, in turn causing reduced niacin sensitivity in depressed patients. Alternatively, it has also been suggested that arachidonic acid-related signaling abnormalities causing a diminished skin response to niacin may be related to disorders of glucose and lipid metabolism in depressed patients [16].

In conclusion, in the present study, the niacin skin flush response was detected in adolescents with depression and healthy controls, and the niacin skin flush response was tentatively found to have potential clinical application in the identification of adolescent depression. However, because the sample size of this study was small, the effects of depression severity and medications were not studied, and the response mechanisms were not explored, the findings need to be considered with caution. In the future, the sample size will be further expanded for in-depth study.

## Data Availability

The datasets generated and/or analysed during the current study are not publicly available because further publications are still being analysed from the data. However, data are available from the corresponding author on reasonable request.

## List of Abbreviations

DSM-5: Diagnostic and Statistical Manual of Mental Disorders, fifth edition
HAMD-24: Hamilton depression scale 24-item
HAMA: Hamilton anxiety scale
ROC: Receiver operating characteristic
AUC: The area under the curve
AA: Arachidonic acid
PLA2: Phospholipase A2
5-HT: 5-hydroxytryptamine

## References

[1] Beauchamp MR, Puterman E, Lubans DR (2018). Physical Inactivity and Mental Health in Late Adolescence. JAMA Psychiatry.

[2] Boers E, Afzali MH, Newton N, Conrod P (2019). Association of Screen Time and depression in adolescence. JAMA Pediatr.

[3] Kieling C, Adewuya A, Fisher HL, Karmacharya R, Kohrt BA, Swartz JR, Mondelli V (2019). Identifying depression early in adolescence. Lancet Child Adolesc Health.

[4] Akingbuwa WA, Hammerschlag AR, Jami ES, Allegrini AG, Karhunen V, Sallis H, Ask H, Askeland RB, Baselmans B, Diemer E (2020). Genetic associations between Childhood Psychopathology and Adult Depression and Associated traits in 42 998 individuals: a Meta-analysis. JAMA Psychiatry.

[5] Wang DD, Hu XW, Jiang J, Sun LY, Qing Y, Yang XH, Gao Y, Cui GP, Li MH, Wang PK (2021). Attenuated and delayed niacin skin flushing in schizophrenia and affective disorders: a potential clinical auxiliary diagnostic marker. Schizophr Res.

[6] Messamore E (2018). The niacin response biomarker as a schizophrenia endophenotype: a status update. Prostaglandins Leukot Essent Fatty Acids.

[7] Karakula-Juchnowicz H, Rog J, Wolszczak P, Jonak K, Stelmach E, Krukow P (2020). SKINREMS-A New Method for Assessment of the Niacin skin flush test response in Schizophrenia. J Clin Med.

[8] Berger GE, Smesny S, Schäfer MR, Milleit B, Langbein K, Hipler UC, Milleit C, Klier CM, Schlögelhofer M, Holub M (2016). Niacin skin sensitivity is increased in adolescents at Ultra-high Risk for psychosis. PLoS ONE.

[9] Singh A, Trumpff C, Genkinger J, Davis A, Spann M, Werner E, Monk C (2017). Micronutrient Dietary Intake in Latina pregnant adolescents and its Association with Level of Depression, stress, and Social Support. Nutrients.

[10] Bosveld-van Haandel L, Knegtering R, Kluiter H, van den Bosch RJ (2006). Niacin skin flushing in Schizophrenic and depressed patients and healthy controls. Psychiatry Res.

[11] Smesny S, Baur K, Rudolph N, Nenadic I, Sauer H (2010). Alterations of niacin skin sensitivity in recurrent unipolar depressive disorder. J Affect Disord.

[12] Ye L, Cao Z, Lai X, Wang W, Guo Z, Yan L, Wang Y, Shi Y, Zhou N (2019). Niacin fine-tunes energy homeostasis through canonical GPR109A signaling. FASEB J.

[13] Ibrahim WW, Sayed RH, Kandil EA, Wadie W (2022). Niacin mitigates blood-brain barrier tight junctional proteins dysregulation and cerebral inflammation in ketamine rat model of psychosis: role of GPR109A receptor. Prog Neuropsychopharmacol Biol Psychiatry.

[14] Qing Y, Liang J, Wang J, Wan C, Ke X (2022). Attenuated niacin skin flushing response in children and adolescents with mental disorders: a transdiagnostic early warning marker. Schizophr Res.

[15] Hu Y, Xu L, Gan R, Wu G, Tang X, Wei Y, Cui H, Hui L, Tang Y, Li C (2022). A potential objective marker in first-episode schizophrenia based on abnormal niacin response. Schizophr Res.

[16] Sun L, Yang X, Jiang J, Hu X, Qing Y, Wang D, Yang T, Yang C, Zhang J, Yang P (2018). Identification of the niacin-blunted subgroup of Schizophrenia patients from Mood disorders and healthy individuals in Chinese Population. Schizophr Bull.

[17] Wang J, Qing Y, Liang J, Cui G, Wang Q, Zhang J, Yang X, Li M, Wang D, Fan Z, Chu K, Zhang J, Ke X, Wan C (2023). Identification of adolescent patients with depression via assessment of the niacin skin flushing response. J Affect Disord.

